# CURIOSITY TOWARDS ROBOT: THE ROLE OF AGE AND PERSONAL RELEVANCE

**DOI:** 10.1093/geroni/igz038.3212

**Published:** 2019-11-08

**Authors:** Li Chu, Helene H Fung

**Affiliations:** 1 The Chinese University of Hong Kong, Shatin, New Territories, Hong Kong; 2 Chinese University of Hong Kong, Hong Kong, Hong Kong

## Abstract

Being curious has various physical, social and psychological benefits. However, theories like the socioemotional selectivity theory suggest that information seeking goals tend to be overshadowed by emotionally meaningful goals with age. Personality and social psychology research also found consistent decline of curiosity in later adulthood. In contrast, selective engagement theory propose that people simply become more selective on where they allocate their cognitive resources as they age. Particularly, older adults tend to invest in things that have personal relevance. Yet, few studies have explored the interaction between age and personal relevance in the context of information seeking tendencies. We conducted a pre-test-post-test experiment with 50 younger (age 19-34) and 50 older adults (age 60-78). Participants were invited to learn about a robot (Vector by Anki) and freely interact with the robot for about 30 minutes. Questionnaires were filled before and after the interaction. Our results confirmed previous findings that older adults showed lower level of trait curiosity than younger adults (F(1, 98)=21.94, p<.001, ΔR2=). However, older adults actually showed higher level of state curiosity towards robot than their younger counterparts (F(1, 96)=21.94, p<.001). Moreover, there was a marginally significant interaction effect of personal relevance (p = .06). Tukey’s post-hoc test revealed that older adults who perceived increased relevance (M=5.39) after the interaction were significantly more curious than younger adults who also perceived increased relevance (M=4.51, p=.02), but there was no significant age difference when they perceived decreased relevance. Present study offers insights on promoting curiosity among older adults.

